# Designing, Developing, and Testing a Chatbot for Parents and Caregivers of Children and Young People With Rheumatological Conditions (the IMPACT Study): Protocol for a Co-Designed Proof-of-Concept Study

**DOI:** 10.2196/57238

**Published:** 2024-04-03

**Authors:** Polly Livermore, Klaudia Kupiec, Lucy R Wedderburn, Andrea Knight, Ameenat L Solebo, Roz Shafran, Glenn Robert, N J Sebire, Faith Gibson, IMPACT Steering Group

**Affiliations:** 1 Rheumatology Department, Great Ormond Street Hospital for Children NHS Foundation Trust London United Kingdom; 2 NIHR Biomedical Research Centre at Great Ormond Street Hospital for Children London United Kingdom; 3 University College London Great Ormond Street Institute of Child Health London United Kingdom; 4 Centre for Adolescent Rheumatology Versus Arthritis at UCL, UCLH and GOSH London United Kingdom; 5 Division of Rheumatology, The Hospital for Sick Children Toronto, ON Canada; 6 Neurosciences and Mental Health Program, SickKids Research Institute Toronto, ON Canada; 7 Temerty Faculty of Medicine, University of Toronto Toronto, ON Canada; 8 Opthamology Department, Great Ormond Street Children's Hospital NHS Foundation Trust London United Kingdom; 9 Kings College London London United Kingdom; 10 School of Health Sciences, University of Surrey Surrey United Kingdom; 11 Director of Research - Nursing and Allied Health, Great Ormond Street Children's Hospital London United Kingdom; 12 See Acknowledgments

**Keywords:** caregivers, chatbot, paediatric rheumatology, parents and caregivers, parents/carers, pediatric, proof-of-concept, quality of life, rheumatology

## Abstract

**Background:**

Pediatric rheumatology is a term that encompasses over 80 conditions affecting different organs and systems. Children and young people with rheumatological chronic conditions are known to have high levels of mental health problems and therefore are at risk of poor health outcomes. Clinical psychologists can help children and young people manage the daily difficulties of living with one of these conditions; however, there are insufficient pediatric psychologists in the United Kingdom. We urgently need to consider other ways of providing early, essential support to improve their current well-being. One way of doing this is to empower parents and caregivers to have more of the answers that their children and young people need to support them further between their hospital appointments.

**Objective:**

The objective of this co-designed proof-of-concept study is to design, develop, and test a chatbot intervention to support parents and caregivers of children and young people with rheumatological conditions.

**Methods:**

This study will explore the needs and views of children and young people with rheumatological conditions, their siblings, parents, and caregivers, as well as health care professionals working in pediatric rheumatology. We will ask approximately 100 participants in focus groups where they think the gaps are in current clinical care and what ideas they have for improving upon them. Creative experience-based co-design workshops will then decide upon top priorities to develop further while informing the appearance, functionality, and practical delivery of a chatbot intervention. Upon completion of a minimum viable product, approximately 100 parents and caregivers will user-test the chatbot intervention in an iterative sprint methodology to determine its worth as a mechanism for support for parents.

**Results:**

A total of 73 children, young people, parents, caregivers, and health care professionals have so far been enrolled in the study, which began in November 2023. The anticipated completion date of the study is April 2026. The data analysis is expected to be completed in January 2026, with the results being published in April 2026.

**Conclusions:**

This study will provide evidence on the accessibility, acceptability, and usability of a chatbot intervention for parents and caregivers of children and young people with rheumatological conditions. If proven useful, it could lead to a future efficacy trial of one of the first chatbot interventions to provide targeted and user-suggested support for parents and caregivers of children with chronic health conditions in health care services. This study is unique in that it will detail the needs and wants of children, young people, siblings, parents, and caregivers to improve the current support given to families living with pediatric rheumatological conditions. It will be conducted across the whole of the United Kingdom for all pediatric rheumatological conditions at all stages of the disease trajectory.

**International Registered Report Identifier (IRRID):**

DERR1-10.2196/57238

## Introduction

### Background

Pediatric rheumatological illnesses are chronic inflammatory conditions that affect the musculoskeletal system [1,2]. While some are rare, juvenile idiopathic arthritis (JIA), the most common rheumatological disease of childhood, currently affects 10,000 children and young people in the United Kingdom [3]. JIA occurs as frequently as juvenile diabetes mellitus and 10 times more frequently than acute lymphoblastic leukemia [4]. Children and young people with rheumatological conditions are well known to experience a high psychological burden, with decreased quality of life, increased pain and disease activity, physical disabilities, school absenteeism, suboptimal medication adherence, and transition challenges [1,5]. Up to two-thirds of young people continue with active disease into adulthood [6,7], with visible and invisible symptoms causing marked psychological ramifications. High levels of mental health comorbidity and consequent risk for adverse health outcomes are common in children and young people with rheumatological conditions [1,8]. The British Society of Paediatric and Adolescent Rheumatology (BSPAR) Standards of Care (2010) include the recommendation that a pediatric clinical psychologist should be part of the core multidisciplinary team [9]. However, a 2021 survey of 15 high-volume pediatric rheumatology UK centers highlighted that 7 centers failed to achieve this [10]. In 2019, the British Society of Rheumatology’s (BSR) pediatric and adolescent state of play report [11] highlighted poor access to psychology expertise and recommended increased psychology provision. Early identification of children and young people who are struggling is known to improve outcomes, but current systems fall short of providing adequate emotional and mental health support to those with rheumatological conditions [12].

A review examining the impact of living with a child with a long-term health condition revealed that parents perceive that they are not always supported in their quest for information, and their ultimate responsibility for their child’s health can be overwhelming [13]. Parents and caregivers described that when information is not available from health care professionals, they are compelled to search for it elsewhere [14] and feel the need to “take charge,” becoming advocates for their child through “tenacious information seeking” [15]. Patient and Public Involvement and Engagement (PPIE) activities revealed that available parental support is currently inequitably distributed and accessed. Charity access is inconsistent, with awareness dependent on clinical teams, other parents, or “internet searching.” Education support offered at “family weekends” is limited by geography and capacity. Social media closed groups are only available to those who know about them, and due to the unregulated content, parents were skeptical about the levels of support received.

Rheumatologists and nurse specialists in the United Kingdom identified a lack of clinic time as one of the biggest limitations to asking about emotional and mental health support in appointments, followed by the absence of resources when concerns are raised [10]. While clinic time is limited, waiting lists are too long, and an increase in the number of psychologists is awaited, upskilling parents has the potential to improve their child’s psychological well-being and prevent the need for later mental health interventions. The James Lind Alliance also supports this in its 10 identified priority areas for mental health in children and young people, specifically: what methods can parents and caregivers use to identify that their child’s mental health is deteriorating and what are the most effective early intervention strategies for supporting them to improve mental resilience [16]. In 2023, 5 UK pediatric rheumatology charities were so worried about mental health that they collaborated to develop and deliver a survey to understand the scale of the problem. The results from this work showed that out of 291 parents and caregivers completing the survey, 218 (82%) parents and caregivers reported their child’s diagnosis as impacting their own mental health. It also highlighted that 60% of children and young people have needed mental health support since diagnosis (had help, are undergoing help, or are on a waiting list), and in relation to condition specific difficulties, 81% needed help with needle phobia, 80% are reluctant to take their rheumatology medicines, and 78% are struggling with side effects from medications [17].

At the time of this study application, chatbots were being used as a means of providing useful information in a variety of settings [18]. Chatbots are typically cloud-based programs that require internet-connected devices, and users interact with them in a number of ways, including through text and voice. In particular, chatbots such as Vincent have proven to be useful to enhance mental health [19], iHelper provides guided self-assessment for stress, anxiety, depression, and self-esteem, and Woebot is a text-based conversational agent providing 2-weeks of self-help, proven to significantly reduce depression [20]. Chatbots are beginning to be used in National Health Service (NHS) services, such as “Ask Olli” for parents at Alderhey [21] and “Oriel Assistant” for patients and staff at Moorfields [22], and evidence showed that most internet users would be receptive to using health chatbots [23]. Since the funding for the study was awarded, chatbot interest has progressed rapidly with the launch of commercially available chatbots. Chatbots potential in health care is also growing, with research progressing in this area. For example, ChatGPT and GPT-4 already show promise in translating radiology reports into plain language for families [24]. The exact deliverable for this study, however, is still work in progress and will be decided by the co-design group, taking into account issues such as digital poverty, scalability, advantages and disadvantages of platforms, data security, and outcome measures.

The “Interventions to improve Mental health supPort in families with children And young people with Chronic rheumaTological conditions” (IMPACT) proof-of-concept study aims to design, develop, and test a chatbot intervention for parents and caregivers of children and young people with rheumatic conditions. It is hoped that by empowering parents and caregivers, we will strengthen the support around children and young people, prevent anxieties and uncertainties from escalating, and improve other aspects of pediatric rheumatological care, such as adherence to therapies. This intervention is not to replace human contact but to be an adjunct when human contact is limited, for example, between appointments and while on holiday.

### Study Setup

The research team is led by the principal investigator (PL), who is a senior pediatric rheumatology nurse, and the research facilitator (KK), who has a psychology degree and background in pediatric rheumatology research. This study proposal was submitted for a personal postdoctoral National Institute for Health and Social Care Research (NIHR) Advanced Clinical Academic Fellowship (ACAF; NIHR 302864), awarded in 2023 to PL, the first and currently (at the time of writing) only ACAF award given to a nurse in the United Kingdom. The study timeline is from April 2023 to April 2026.

### Patient and Public Involvement and Engagement

A strong and engaged UK-wide steering group of children, young people, parents, caregivers, and health professionals is pivotal to the success of this study. The group is composed of 4 children and young people, 4 parents and caregivers, and 3 health care professionals (a clinical psychologist in pediatric rheumatology, a senior pediatric nurse working in rheumatology research, and a clinical informatics expert). The working group currently includes over 30 children and young people, parents and caregivers, health professionals, and key charity stakeholders who have all been involved in the study proposal since idea conception.

PPIE activities began over 2 years ago and helped shape this study. Initially, children, young people, parents, caregivers, and health professionals were asked whether they needed more support in living with a chronic pediatric rheumatological condition. As conversations proved there was a need to increase support to improve psychosocial well-being, discussions moved over time to consider what this may look like. Initially, the intervention was expected to be tailored toward children and young people; however, as conversations continued, parents and caregivers were open about their need for further support for themselves. A digital intervention was suggested that could help to better prepare their child through their “firsts” (first joint injection, first blood test, or first scan), know the right questions to ask in appointments, know how to have conversations with school, know how to encourage medication adherence, know how to talk about distorted body image due to corticosteroid therapy, know how to encourage home exercises, and know how to identify red flags in their child’s mental health and where to go for help. During brainstorming activities, the idea of a “chatbot” was presented by a parent and unanimously selected as a potential targeted intervention. Parents and caregivers particularly liked the anonymity of chatbots and their constant availability.

Regular meetings, both web-based and face-to-face, have ensured key decisions have been agreed upon as a group, for example. whether 2 participants from the same family could be included in the focus groups. Key documentation has been reviewed by both the steering and working groups, and they will continue to be involved from the beginning to the end of the study. The methodology used for this study ensures that the study is directed by children, young people, parents, and caregivers, ultimately for children, young people, parents, and caregivers.

## Methods

### Methodological Underpinnings

This research study uses experience-based co-design (EBCD) methodology to guide the project, underpinned by the Medical Research Council (MRC)-NIHR Complex Intervention Research Framework [25]. EBCD is a form of participatory research that combines user-centered design and learning theory and is delivered through a 6-stage collaborative process (Textbox 1) [26,27]. These stages will be embedded throughout the study, maximizing the potential to use creativity and ideation to generate wide-ranging ideas and maximize opportunities for innovation, as recommended in the recently updated research framework [25].

**The 6 stages of experience-based co-design (EBCD).**

**Stage:**

- Project set-up
- Staff experiences
- Patient and caregiver experience
- Feedback and co-design
- Co-design teams
- Celebration event

A “trigger film” (short film) is integral to the EBCD methodology and highlights “touch points,” which elicit shared reflections. For this study, the focus groups and planned exercises during the workshops will be video recorded, then edited to produce the trigger film. This film is useful to understand some of the key decisions made throughout the study and can be a useful aid for dissemination. Using the EBCD methodology is compliant with the NIHR Participant in Research Experience survey, which recommends that “technology provided to participants should be tested for reliability and ease of use and co-designed with the intended users” [28].

### Aim

This proof-of-concept study aims to design, develop, and test a chatbot intervention to provide enhanced support compared to current clinical practice that is accessible, acceptable, and usable for parents and caregivers.

### Inclusion and Exclusion Criteria

Children and young people aged 8 years or older with a rheumatological condition diagnosed before the age of 17 years and their siblings will be invited to join focus groups. Parents and caregivers of those with rheumatological conditions will also be invited to parent-specific focus groups. Health professionals who care for pediatric and adolescent rheumatological patients will also be invited to join health care–specific focus groups. Those without rheumatological conditions as a child or young person, siblings, or parents of these will not be included. Members of our steering group are also excluded from participating as research participants in the focus groups, although they are welcomed as facilitators.

### Designing EBCD Focus Groups

The “core elements” of the revised MRC-NIHR Framework [25] state that it is imperative to understand key uncertainties, consider the context, and engage stakeholders. Therefore, the aim is to understand, from the perspective of children, young people, siblings, parents, caregivers, and health professionals, what support they think would be useful and whether they think this could be delivered within a chatbot intervention. Focus groups will be used to yield rich qualitative data from a range of individuals from across the United Kingdom. A minimum of 8 focus groups are planned, with approximately 6-12 participants in each group. The parent and caregiver groups will outnumber the child or young person, sibling, and health care professional groups as the intervention is ultimately for parents and caregivers. However, we are also interested in asking participants with rheumatic conditions what they would have found helpful for their parents or caregivers to know, asking siblings for their perspectives, and asking health professionals to discuss where they see more support being offered. Groups will be undertaken remotely or face-to-face, depending on the requests of the majority of participants (in line with NIHR PPIE survey findings) [29].

Parents and caregivers, siblings, children, and young people will be recruited using consecutive sampling. Participants will learn about the study through study advertisement flyers in clinical settings or by being given a flyer. Ethical approval has been granted for local pediatric and adolescent rheumatology centers to display and distribute flyers, and in conjunction with pediatric rheumatology charity social media channels and email lists, this should increase the opportunities for inclusivity across all 4 nations of the United Kingdom. A study-specific website has been developed that encourages interested children, young people, or parents and caregivers to contact the study team to find out more, or if they are unable to access technology, their local team can contact the IMPACT research team on their behalf. Language-specific study documentation and interviews will be offered for those for whom English is not their first language. Staff will be recruited by seeing the study flyers and contacting the study team. A sampling matrix will ensure representation from professional groups and centers.

Consent will be sought for audio and visual recording, the former to guide data analysis, while the video recording will form the trigger film. Thematic analysis [30] will be conducted by 2 members of the research team and discussed with the steering group to identify themes critical to shaping chatbot development using NVivo (QSR International). The final themes will help develop the chatbot.

### Developing EBCD Workshops

The working group will meet in 2 face-to-face workshops to discuss the themes and help identify the core components of the chatbot intervention. The workshops will use personas and scenarios developed from the focus group discussion to help inform the chatbot development process. Such creative and participatory methods will allow the shared experiences of the members of the working group to shape the intervention development process. The workshop will be video recorded as recommended in the EBCD methodology, with excerpts of the film used later in the trigger film.

Over the last few years, as chatbot technology has developed at an alarming rate, with large language models now leading the way, their popularity has increased [31]. While it is not possible to be specific regarding the content of the chatbot until the focus groups have been completed and analyzed, a content management system will be developed to inform the chatbot development. It is anticipated that the chatbot may include such functions as (1) frequently asked questions, (2) rheumatology-specific information, (3) information about managing “firsts,” (4) parental red-flag identification and signposting, and (5) persuasive argument roleplay. Using agile principles, the exact technologies and approaches used may change as the team learns more about the user requirements. Development of the chatbot will occur within existing recommendations such as the National Institute for Health and Care Excellence Evidence Standards Framework for Digital Health Technologies [32], NHS Digital guidance for cloud security [33], and the Department of Health guidelines for “Putting data, digital, and tech at the heart of transforming the NHS” [34].

### User Testing the Agile Sprint Methodology

Chatbots can potentially lead to frustration, anger, dissatisfaction, and, at worst, disengagement with the technology if not designed and developed with key stakeholders [35,36]. This is not a new technology; however, using it for this purpose for parents and caregivers of children and young people with rheumatological conditions is new. Therefore, the crucial step before a larger study must be to determine accessibility, acceptability, and usability through user testing.

User testing will occur through an iterative methodology with short product development cycles and the deployment of the prototype to groups of parents and caregivers. This will ensure user feedback directs incremental iterative software development. This process of “agile development” runs in a cycle of design, develop, test, and refine (termed a “sprint”). Every 3-month cycle, we will user-test, analyze, and develop. At least 25 parents and caregivers will be recruited for each cycle, which is anticipated to be up to 4 cycles in total. While the majority of participants will be naïve to each cycle, some may be invited back to ‘test’ new modifications in the next cycle. Parents and caregivers will be requested to use the chatbot on a number of occasions for a defined frequency of time.

Eligible parents and caregivers will be those of a child or young person who was diagnosed while being aged 17 years or younger with a chronic pediatric rheumatological condition and who agrees to complete the user metrics throughout the testing period. Flyers in local hospitals will again advertise this part of the study to parents and caregivers, who will then contact the study team for further information. If families are identified as those who do not have access to technology, individual conversations will occur to investigate whether a device may be loaned. All parents and caregivers will be sent an information pack and must provide written consent. Of note, the chatbot technology can integrate “Google Translate,” and as such, families who do not have English as a first language will be able to participate.

Usability outcome measures and qualitative experiences will be sought in order to understand the acceptability of the chatbot for progression to a future study. Outcome measure selection will be informed by exploring what success would look like for families. These may include such measures as (1) attrition of participants; (2) engagement and duration of conversations; (3) user satisfaction measures, such as the System Usability Scale (SUS) [37,38], the User Experience Questionnaire (UEQ) [37], and the Net Promoter Score (NPS) [39,40]; and (4) semistructured interviews with the final group of “sprint” participants.

### Ethical Considerations

The study will be conducted in accordance with the Principles of Good Clinical Practice and the Declaration of Helsinki. Ethical approval from the Health Research Authority has been received for the study (approval received on August 31, 2023, from the Yorkshire & The Humber–Leeds West Research Ethics Committee, IRAS ID: 329476. REC Reference:23/YH/0172). All participants are asked to provide informed written consent or assent (for those aged 16 years or younger, paired with parental or caregiver’s consent) before being enrolled in the study. Consent and assent are requested to audio and video record the focus groups and workshops to enable data analysis and the production of the trigger film, while also requesting to use anonymized quotes and pictures of physical creations in dissemination.

## Results

The study is ongoing. As of February 28, 2024, we have enrolled 73 children, young people, parents, caregivers, and health professionals in 12 focus groups so far. We have had over 280 children, young people, siblings, parents, and caregivers reach out to be included in focus groups, and therefore an ethics amendment has been sought to increase our recruitment target. This huge interest validates the need for the study, and already interesting ideas are emerging. Preliminary results will be published from the focus groups by the end of 2024 and from the user testing by the beginning of 2026.

## Discussion

The rheumatological conditions of childhood affect the whole family. Children and young people have been shown to suffer with their emotional and mental health as they cope with the implications of a chronic health condition. Parents, caregivers, and siblings of the child have also been shown to experience difficulties as they navigate the new normal family lifestyle and the implications of the health condition on their own lives. Parents and caregivers report that for conditions such as JIA, there is a wealth of information available on the internet; however, it is not always clear how robust and trustworthy this information is, while for rarer pediatric rheumatological conditions, there is much less available information, and searching for what little there is can be upsetting and often futile. Providing information and support in the style of a chatbot has many advantages, including physician-ratified and endorsed information, anonymity, being accessible throughout the day and night, and the ability to integrate new novel functions, such as the ability to practice difficult conversations with the chatbot enacting as their child or young person. Therefore, the aim of this study is to investigate whether a chatbot intervention could provide additional support to families between appointments without the need for human resources.

If the chatbot proves to be a success, then such an intervention may be useful in other diseases and with other populations. If the chatbot shows that parents and caregivers do not find it useful and its function is limited, this is useful learning in the current rapidly advancing technological climate, and further intervention and development may be commissioned. If the intervention is deemed acceptable, then to draw conclusions regarding the effectiveness of the intervention, a further study would be required. Discussions will begin early to scope out options for embedding the chatbot into NHS services in order to enhance the transition from research to service delivery following further assessment. Links to research outputs will be made available on the IMPACT study website [41].

This study has several limitations; primarily, as a proof-of-concept study, it will be difficult to generalize the findings. Coping with a chronic health condition varies on a daily basis depending on disease severity, treatments and their side effects, social support, and daily mood fluctuations. Therefore, the chatbot may prove useful to families for the short duration of this study, but as there is no external control group, analyzing all variables and drawing sound conclusions could be challenging. Also, due to the technological nature of a chatbot intervention, families who do not have easy access to technology may be disadvantaged; however, we are in the process of scoping local technology resources, which can be loaned out if required. We also set a lower age limit (aged 8 years or older) for child participation. This was to be mindful that the questions we are asking children about what else they would have liked support with would be difficult for most children aged 8 years or younger to answer. However, we are aware that in some instances, parents and caregivers feel their younger child may have liked the opportunity to take part.

We believe this study can have significant future applications and implications, such as learning from family perspectives on how we can improve upon current rheumatological clinical care, providing a responsive and tailored intervention to help support families better, and an understanding of how such technology could be embedded into the wider health care system. This is particularly relevant in today’s current climate of limited staffing resources and an interest in delivering care differently. This study also offers an insight into how a chatbot could be used for families who live at a distance from their health care site, thus offering advantages over face-to-face support appointments and providing a resource for families, which may in turn lessen the need to contact the local team for support and thus free up valuable resources. The interest in the study so far, at over 280 families who have reached out in just over three months, over double the planned recruitment for the focus groups, is clear evidence that we need to do more to listen to, engage with, and support families further.

## Appendix

Multimedia Appendix 1 Reviewer comments.

## Abbreviations

ACAF: Advanced Clinical Academic Fellowship
BSPAR: British Society of Paediatric and Adolescent Rheumatology
BSR: British Society of Rheumatology
EBCD: experience-based co-design
IMPACT: Interventions to improve Mental health supPort in families with children And young people with Chronic rheumaTological conditions
JIA: juvenile idiopathic arthritis
MRC: Medical Research Council
NHS: National Health Service
NIHR: National Institute for Health and Social Care Research
NPS: Net Promoter Score
PPIE: Patient and Public Involvement and Engagement
SUS: System Usability Scale
UEQ: User Experience Questionnaire

## Data Availability

The anonymized data sets analyzed during this study will be available from the corresponding author upon reasonable request.
