# Supplementary material for: Designing, Developing, and Testing a Chatbot for Parents and Caregivers of Children and Young People With Rheumatological Conditions (the IMPACT Study): Protocol for a Co-Designed Proof-of-Concept Study
Source: JMIR Res Protoc. 2024 Apr 3;13:e57238. doi: 10.2196/57238 (PMC11024752; doi:10.2196/57238)
Supplement: Multimedia Appendix 1 [file resprot_v13i1e57238_app1.pdf]

NIHR | National Institute for Health and Care Research

Summary

**Reference Number**	NIHR302864
**Lead Applicant**	Dr Polly Livermore
**Research Title**	IMPACT : Interventions to improve Mental health Provision in children And young people with Chronic rheumaTological conditions
**Total Research Cost**	604,985.00

Our purpose for collecting this data is to communicate with you about your application and have the necessary information to evaluate you for a grant. The data we collect here is collected in the public interest. Information provided here may be subject to Freedom of Information requests.

The NIHR Academy is part of the Department for Health and Social Care (DHSC), National Institute for Health and Care Research (NIHR). The contracting agent for NIHR Academy is the Leeds Teaching Hospital Trust (LTHT). The DHSC is the Data Controller and LTHT is the Data Processor under the General Data Protection Regulation (GDPR) EC 2016/679. DHSC NIHR respects the privacy of individuals who share their data and processes it in a manner that meets the requirements of GDPR. The DHSC Data Protection Officer can be contacted by email at: data_protection@dhsc.gov.uk

The NIHR privacy policy includes further information including ways we may use your data, our contact details and details on your individual rights regarding how your data is used. Your data may be shared across the NIHR, including with other coordinating centres, to allow the application to be managed and for statistical analysis, and with external grant reviewers as part of the process for managing the allocation of a grant. This notice is under constant review and will be updated and / or revised based on that review as appropriate.

Table Of Contents

1. ICA CL & SCL PPI Assessment Form

1. ICA CL & SCL PPI Assessment Form

Plain English Summary Assessment Form

This assessment will be provided as feedback to the applicant.

You will not be able to submit the form until you have confirmed your agreement to abide by the Code of Practice, graded the Plain English Summary as 'Satisfactory' or 'Unsatisfactory', and added comments that justify your grading within the free-text box.
A Plain English Summary is a brief summary of a research project or research proposal that has been written for members of the public, rather than for researchers or professionals. It should be written in plain English, avoiding the use of jargon and explaining any technical terms that have to be included. The summary should explain why the research is being suggested, what the researchers aim to achieve, and how this may impact on the field(s) of interest.

If the project is funded, the Plain English Summary will be published as stand-alone descriptive text on a variety of websites, and, as such, needs to serve as a description of the work in full.

**Standard of Plain English Summary**	Satisfactory

**Justification of grading provided above**<br>Please use the space provided to explain your grading, highlighting strengths and weaknesses of the summary as appropriate.
This is a very well written PES. It is well structured with clear headings and, although it contains technical medical and technological concepts, it explains them well. The 'why / what / how' and impact are all explained clearly.<br>It really benefits from not trying to explain everything that will be done which is where PESs tend to fall down.<br>The only thing that I'm not sure about is in the background where it describes how the study would 'strengthen networks'. I think this means something quite specific to people and I'm not sure that's really what the study offers. Other than that, excellent.

NIHR | National Institute for Health and Care Research

Summary

**Reference Number**	NIHR302864
**Lead Applicant**	Dr Polly Livermore
**Research Title**	IMPACT : Interventions to improve Mental health Provision in children And young people with Chronic rheumaTological conditions
**Total Research Cost**	604,985.00

Our purpose for collecting this data is to communicate with you about your application and have the necessary information to evaluate you for a grant. The data we collect here is collected in the public interest. Information provided here may be subject to Freedom of Information requests.

The NIHR Academy is part of the Department for Health and Social Care (DHSC), National Institute for Health and Care Research (NIHR). The contracting agent for NIHR Academy is the Leeds Teaching Hospital Trust (LTHT). The DHSC is the Data Controller and LTHT is the Data Processor under the General Data Protection Regulation (GDPR) EC 2016/679. DHSC NIHR respects the privacy of individuals who share their data and processes it in a manner that meets the requirements of GDPR. The DHSC Data Protection Officer can be contacted by email at: data_protection@dhsc.gov.uk

The NIHR privacy policy includes further information including ways we may use your data, our contact details and details on your individual rights regarding how your data is used. Your data may be shared across the NIHR, including with other coordinating centres, to allow the application to be managed and for statistical analysis, and with external grant reviewers as part of the process for managing the allocation of a grant. This notice is under constant review and will be updated and / or revised based on that review as appropriate.

Table Of Contents

1. ICA CL & SCL PPI Assessment Form

1. ICA CL & SCL PPI Assessment Form

Plain English Summary Assessment Form

This assessment will be provided as feedback to the applicant.

You will not be able to submit the form until you have confirmed your agreement to abide by the Code of Practice, graded the Plain English Summary as 'Satisfactory' or 'Unsatisfactory', and added comments that justify your grading within the free-text box.
A Plain English Summary is a brief summary of a research project or research proposal that has been written for members of the public, rather than for researchers or professionals. It should be written in plain English, avoiding the use of jargon and explaining any technical terms that have to be included. The summary should explain why the research is being suggested, what the researchers aim to achieve, and how this may impact on the field(s) of interest.

If the project is funded, the Plain English Summary will be published as stand-alone descriptive text on a variety of websites, and, as such, needs to serve as a description of the work in full.

**Standard of Plain English Summary**	Satisfactory

**Justification of grading provided above**<br>Please use the space provided to explain your grading, highlighting strengths and weaknesses of the summary as appropriate.
Clear and well-structured summary. Explains the background, rationale for the study and the main activities.<br><br>Minor style point - the repeated use of the acronym CYP is clunky and detracts from the flow - ideally remove some of them.

NIHR | National Institute for Health and Care Research

Summary

**Reference Number**	NIHR302864
**Lead Applicant**	Dr Polly Livermore
**Research Title**	IMPACT : Interventions to improve Mental health Provision in children And young people with Chronic rheumaTological conditions

Our purpose for collecting this data is to communicate with you about your application and have the necessary information to evaluate you for a grant. The data we collect here is collected in the public interest. Information provided here may be subject to Freedom of Information requests.

The NIHR Academy is part of the Department for Health and Social Care (DHSC), National Institute for Health and Care Research (NIHR). The contracting agent for NIHR Academy is the Leeds Teaching Hospital Trust (LTHT). The DHSC is the Data Controller and LTHT is the Data Processor under the General Data Protection Regulation (GDPR) EC 2016/679. DHSC NIHR respects the privacy of individuals who share their data and processes it in a manner that meets the requirements of GDPR. The DHSC Data Protection Officer can be contacted by email at: data_protection@dhsc.gov.uk

The NIHR privacy policy includes further information including ways we may use your data, our contact details and details on your individual rights regarding how your data is used. Your data may be shared across the NIHR, including with other coordinating centres, to allow the application to be managed and for statistical analysis, and with external grant reviewers as part of the process for managing the allocation of a grant. This notice is under constant review and will be updated and / or revised based on that review as appropriate.

Table Of Contents

1. Review

1. Review

Selection Committee Assessment

Please read the application and:

1. For each of the listed assessment criteria, please select (strong/average/uncompetitive) that best represents your opinion of the application.
2. For each section please provide a score using the following scale:
   5: EXCELLENT 4: VERY GOOD 3: SATISFACTORY 2: UNCOMPETITIVE 1: POOR
3. At the end of the form provide an overall recommendation using the grading system described at the end.

Please add specific comments in the boxes provided to accompany your scores. These comments will be used directly as feedback to the applicant and we ask that you bear this in mind when completing the comments.

Applicant

The quality and relevance of the applicant's research experience and achievements to the undertaking of the research described within the application.
Average

High quality outputs from previous research experience and training relative to career stage and background.
Strong

The relevance of the applicant's recent and overall professional experience to the development of a practitioner academic career in the proposed area.
Strong

On a trajectory to become a future health/social care practitioner academic leader:<br>**-** For doctoral and early career post-doctoral applicants or those seeking to re-establish their research career - evidence of commitment to a career as a practitioner academic.<br>- For more experienced post-doctoral applicants; evidence of independence or starting to establish independence as a researcher, including establishing collaborations and building research capacity.
Strong

**Score (1-5) 5: EXCELLENT 4: VERY GOOD 3: SATISFACTORY 2: UNCOMPETITIVE 1: POOR**
4

**Comments**
Strong candidate with a clear track of clinical academic awards for progression, decent set of papers including first author (NB PhD) however no identified example of co-investigator of significant grants included in the application, which will be an area to develop alongside a post-doctoral award. Has potential to be a leader in their field and grow capacity.

Proposed Research

The suitability of the proposed research programme to the level of award and scope of funding.
Strong

The quality of the research design and its suitability to answer the research question being proposed.
Strong

The potential of the project to benefit patients/service users, carers, the public or health and/or care services.
Strong

The extent to which the involvement of patients/service users, carers and the public is appropriate and meaningful.
Strong

The quality, scope and relevance of the review of existing evidence.	Strong

Quality of the plain English summary.	Average

The capacity of the proposed project to serve as a vehicle for academic and professional development.
Strong

The amount of support requested (both financial and duration of the Fellowship) is justified and evidence based.
Strong

**Score (1-5) 5: EXCELLENT 4: VERY GOOD 3: SATISFACTORY 2: UNCOMPETITIVE 1: POOR**
4

**Comments**
Strong co-production in the development and testing of the intervention, and clear rationale for why it is needed.<br><br>It is not clear from the information provided how the candidate would address equality diversity and inclusion.<br><br>It is not clear from the application that there is a definitive answer as to whether this digital intervention is effective however, an effectiveness trial is beyond the scope of the fellowship.

Sites, Training and Support

The extent to which the statement of support is tailored to the applicant and demonstrates a partnership of the Host and Partner Organisations to support the applicant's Practitioner Academic career development.
Average

Evidence of the host organisations’ commitment to creating and maintaining an inclusive and supportive research culture, including evidence of commitment to the principles of equality, diversity and inclusion and research integrity.
Average

Evidence of the Host Organisations’ support and commitment to other early career researchers.
Average

The track record of academic department in the research area being proposed.	Strong

The suitability of the proposed training to the needs of the project.	Average

The extent to which the proposed practice activities will support the development of the applicant as a practitioner academic.
Average

The extent to which the training and development plan will support the practitioner academic career aspirations of the applicant.
Average

The suitability and experience of the proposed academic support team, and their appropriateness to the development of the applicant's practitioner academic career.
Strong

The suitability and experience of the proposed practice support team, and their appropriateness to the development of the applicant's practitioner academic career.
Strong

**Score (1-5) 5: EXCELLENT 4: VERY GOOD 3: SATISFACTORY 2: UNCOMPETITIVE 1: POOR**
3

**Comments**
The supporting statement would be strengthened through greater clarity and detail on the role of the applicant in the future.<br><br>Given that the next step would be to lead significant grant applications, more detail around developing this experience would strengthen the application.

**Significant issues that need clarifying at interview (if relevant)**
How will you address equality diversity and inclusion in your study in both ensuring the digital tool meets a diverse set of needs, as well as ensuring diversity in the PPIE?<br><br>What are your plans to develop experience and skills in working with collaborators on significant grant applications and project leadership in the future?

Please complete the following using the Grading system described below

Grade	Criteria	Descriptor	Outcome
A	Fully meets the criteria in all areas assessed	Excellent candidate and application	Interview / Shortlist

B	Meets all the assessment criteria with minor weaknesses	Very good candidate and application	Interview / Shortlist
C	Reasonable quality applicant however does not adequately meet the criteria	Reasonable candidate and application	Reject
D	Not competitive	Poor candidate and application	Reject

**Grade**	B

NIHR | National Institute for Health and Care Research

Summary

**Reference Number**	NIHR302864
**Lead Applicant**	Dr Polly Livermore
**Research Title**	IMPACT : Interventions to improve Mental health Provision in children And young people with Chronic rheumaTological conditions

Our purpose for collecting this data is to communicate with you about your application and have the necessary information to evaluate you for a grant. The data we collect here is collected in the public interest. Information provided here may be subject to Freedom of Information requests.

The NIHR Academy is part of the Department for Health and Social Care (DHSC), National Institute for Health and Care Research (NIHR). The contracting agent for NIHR Academy is the Leeds Teaching Hospital Trust (LTHT). The DHSC is the Data Controller and LTHT is the Data Processor under the General Data Protection Regulation (GDPR) EC 2016/679. DHSC NIHR respects the privacy of individuals who share their data and processes it in a manner that meets the requirements of GDPR. The DHSC Data Protection Officer can be contacted by email at: data_protection@dhsc.gov.uk

The NIHR privacy policy includes further information including ways we may use your data, our contact details and details on your individual rights regarding how your data is used. Your data may be shared across the NIHR, including with other coordinating centres, to allow the application to be managed and for statistical analysis, and with external grant reviewers as part of the process for managing the allocation of a grant. This notice is under constant review and will be updated and / or revised based on that review as appropriate.

Table Of Contents

1. Review

1. Review

Selection Committee Assessment 2

Please read the application and:

1. For each of the listed assessment criteria, please select (strong/average/uncompetitive) that best represents your opinion of the application.
2. For each section please provide a score using the following scale:
   5: EXCELLENT 4: VERY GOOD 3: SATISFACTORY 2: UNCOMPETITIVE 1: POOR
3. At the end of the form provide an overall recommendation using the grading system described at the end.

Please add specific comments in the boxes provided to accompany your scores. These comments will be used directly as feedback to the applicant and we ask that you bear this in mind when completing the comments.

Applicant

The quality and relevance of the applicant's research experience and achievements to the undertaking of the research described within the application.
Average

High quality outputs from previous research experience and training relative to career stage and background.
Average

The relevance of the applicant's recent and overall professional experience to the development of a practitioner academic career in the proposed area.
Strong

On a trajectory to become a future health/social care practitioner academic leader:<br>**-** For doctoral and early career post-doctoral applicants or those seeking to re-establish their research career - evidence of commitment to a career as a practitioner academic.<br>- For more experienced post-doctoral applicants; evidence of independence or starting to establish independence as a researcher, including establishing collaborations and building research capacity.
Strong

**Score (1-5) 5: EXCELLENT 4: VERY GOOD 3: SATISFACTORY 2: UNCOMPETITIVE 1: POOR**
4

**Comments**
The applicant has an extensive CV with a track record of advanced clinical activity in the field of paediatric rheumatology. They have received a number of research awards over several years but these are mostly small support grants, and a number of 1st author papers as well as co-authorship on publications. This applications builds on a successful NIHR CDRF, a Health-Education-England (HEE) post-doctoral bridging programme and a NIHR Development-and-Skills-Award (DSE). These awards have led to an acceleration in the applicant's research career and there is clear evidence of using this investment to consolidate a national reputation and build research capacity.

Proposed Research

The suitability of the proposed research programme to the level of award and scope of funding.

Average

The quality of the research design and its suitability to answer the research question being proposed.
Average

The potential of the project to benefit patients/service users, carers, the public or health and/or care services.
Strong

The extent to which the involvement of patients/service users, carers and the public is appropriate and meaningful.
Strong

The quality, scope and relevance of the review of existing evidence.	Strong

Quality of the plain English summary.	Strong

The capacity of the proposed project to serve as a vehicle for academic and professional development.
Average

The amount of support requested (both financial and duration of the Fellowship) is justified and evidence based.
Strong

**Score (1-5) 5: EXCELLENT 4: VERY GOOD 3: SATISFACTORY 2: UNCOMPETITIVE 1: POOR**
4

Comments

The applicant proposes a pilot study completed full time over 3 years - based on the information provided this appears feasible but this will involve design of the intervention from scratch and a number of iterative stages of intervention development and testing - the co-design aspects are critical but also can create a risk in terms of meeting the timelines and milestones. Overall each WP is extensive and maybe overambitious to complete in 3 years given the training and development demands and time involved.

However the proposal to create a digital service for parents supporting their CYP at home, is novel and unique. Role playing and practicing difficult conversations has the potential to provide a different and engaging form of support for parents. If the Intelligent-Virtual-Assistant (IVA) is successful it could transform the experience for parents managing ongoing care for CYP. The staged approach is necessary, and authentic co-design to develop the intervention is critical if parents are the ultimate end users- and this could have wider benefit across the NHS.

The applicant explains the necessary change through PPIE to focus on parents of CYP as the ultimate beneficiaries- this fact that this idea came apparent and unanimously selected as a potential targeted intervention justifies and supports the need for the project.

The choice of EBCD is appropriate but it is not completely clear from the application which elements will be applied- the applicant states a trigger film will be used in WP3 but it is not clear from the information provided why the 6-stages of EBCD cannot provide the overarching approach to co-designing the content, language and activities etc.

It is also not clear from the application why 8 focus groups are necessary - the data generated from each FG is

substantial and the applicant aims to complete these and a systematic review in one year. This stage is also required to progress into WP3,4 and 5.<br><br>The plain English summary is clear and PPIE has been given careful consideration.

Sites, Training and Support

The extent to which the statement of support is tailored to the applicant and demonstrates a partnership of the Host and Partner Organisations to support the applicant's Practitioner Academic career development.
Strong

Evidence of the host organisations' commitment to creating and maintaining an inclusive and supportive research culture, including evidence of commitment to the principles of equality, diversity and inclusion and research integrity.
Strong

Evidence of the Host Organisations' support and commitment to other early career researchers.
Strong

The track record of academic department in the research area being proposed.	Strong

The suitability of the proposed training to the needs of the project.	Strong

The extent to which the proposed practice activities will support the development of the applicant as a practitioner academic.
Average

The extent to which the training and development plan will support the practitioner academic career aspirations of the applicant.
Average

The suitability and experience of the proposed academic support team, and their appropriateness to the development of the applicant's practitioner academic career.
Strong

The suitability and experience of the proposed practice support team, and their appropriateness to the development of the applicant's practitioner academic career.
Strong

**Score (1-5) 5: EXCELLENT 4: VERY GOOD 3: SATISFACTORY 2: UNCOMPETITIVE 1: POOR**
4

**Comments**

Both the academic and practice support team are excellent and bring a range of experiences relevant to the applicant and the proposed research. The training plan is suitable and not too ambitious given the amount of research activities planned within the 3 years, this is combined with visits to institutes of excellence and shadowing clinical mentors.

The host statement is tailored to the applicant and this area of research in collaboration with an industry partner appears well supported. There is a long standing relationship with the host - as the applicant is a well respected leader in their clinical field and the host organisation has a strong track record of work leading research - despite this reputation this appears to be a new and exciting area for both GOS UCL and NHS- and the statement appears to give full support.

Significant issues that need clarifying at interview (if relevant)

The only challenges noted are the ambitious nature of the research and whether the number of work packages are feasible within 3 years. Although support staff are costed for- the balance of work between the lead applicant and the research staff should be explored especially given the time required to complete training and mentoring activities during the fellowship.

Please complete the following using the Grading system described below

Grade	Criteria	Descriptor	Outcome
A	Fully meets the criteria in all areas assessed	Excellent candidate and application	Interview / Shortlist
B	Meets all the assessment criteria with minor weaknesses	Very good candidate and application	Interview / Shortlist
C	Reasonable quality applicant however does not adequately meet the criteria	Reasonable candidate and application	Reject
D	Not competitive	Poor candidate and application	Reject

**Grade**	B

NIHR | National Institute for Health and Care Research

Summary

**Reference Number**	NIHR302864
**Lead Applicant**	Dr Polly Livermore
**Research Title**	IMPACT : Interventions to improve Mental health Provision in children And young people with Chronic rheumaTological conditions

Our purpose for collecting this data is to communicate with you about your application and have the necessary information to evaluate you for a grant. The data we collect here is collected in the public interest. Information provided here may be subject to Freedom of Information requests.

The NIHR Academy is part of the Department for Health and Social Care (DHSC), National Institute for Health and Care Research (NIHR). The contracting agent for NIHR Academy is the Leeds Teaching Hospital Trust (LTHT). The DHSC is the Data Controller and LTHT is the Data Processor under the General Data Protection Regulation (GDPR) EC 2016/679. DHSC NIHR respects the privacy of individuals who share their data and processes it in a manner that meets the requirements of GDPR. The DHSC Data Protection Officer can be contacted by email at: data_protection@dhsc.gov.uk

The NIHR privacy policy includes further information including ways we may use your data, our contact details and details on your individual rights regarding how your data is used. Your data may be shared across the NIHR, including with other coordinating centres, to allow the application to be managed and for statistical analysis, and with external grant reviewers as part of the process for managing the allocation of a grant. This notice is under constant review and will be updated and / or revised based on that review as appropriate.

Table Of Contents

1. Review

1. Review

Selection Committee Assessment 3

Please read the application and:

1. For each of the listed assessment criteria, please select (strong/average/uncompetitive) that best represents your opinion of the application.
2. For each section please provide a score using the following scale:
   5: EXCELLENT 4: VERY GOOD 3: SATISFACTORY 2: UNCOMPETITIVE 1: POOR
3. At the end of the form provide an overall recommendation using the grading system described at the end.

Please add specific comments in the boxes provided to accompany your scores. These comments will be used directly as feedback to the applicant and we ask that you bear this in mind when completing the comments.

Applicant

The quality and relevance of the applicant's research experience and achievements to the undertaking of the research described within the application.
Strong

High quality outputs from previous research experience and training relative to career stage and background.
Strong

The relevance of the applicant's recent and overall professional experience to the development of a practitioner academic career in the proposed area.
Strong

On a trajectory to become a future health/social care practitioner academic leader:<br>- For doctoral and early career post-doctoral applicants or those seeking to re-establish their research career - evidence of commitment to a career as a practitioner academic.<br>- For more experienced post-doctoral applicants; evidence of independence or starting to establish independence as a researcher, including establishing collaborations and building research capacity.
Strong

**Score (1-5) 5: EXCELLENT 4: VERY GOOD 3: SATISFACTORY 2: UNCOMPETITIVE 1: POOR**
5

**Comments**
The applicant has a strong track record in the field and the information provided in the application indicates they are knowledgeable about the issue and thus lends confidence that they are well placed to conduct such research.

Proposed Research

The suitability of the proposed research programme to the level of award and scope of funding.
Strong

The quality of the research design and its suitability to answer the research question being proposed.

Strong

The potential of the project to benefit patients/service users, carers, the public or health and/or care services.
Strong

The extent to which the involvement of patients/service users, carers and the public is appropriate and meaningful.
Strong

The quality, scope and relevance of the review of existing evidence.	Strong

Quality of the plain English summary.	Strong

The capacity of the proposed project to serve as a vehicle for academic and professional development.
Strong

The amount of support requested (both financial and duration of the Fellowship) is justified and evidence based.
Strong

**Score (1-5) 5: EXCELLENT 4: VERY GOOD 3: SATISFACTORY 2: UNCOMPETITIVE 1: POOR**
5

**Comments**
The proposal is strong and well developed and the result of the study will have a great potential and usefulness for health care professionals as well as CP and their parents.<br><br>The application clearly highlights the quality of proposed study, and includes a good level of detail.

Sites, Training and Support

The extent to which the statement of support is tailored to the applicant and demonstrates a partnership of the Host and Partner Organisations to support the applicant's Practitioner Academic career development.
Strong

Evidence of the host organisations' commitment to creating and maintaining an inclusive and supportive research culture, including evidence of commitment to the principles of equality, diversity and inclusion and research integrity.
Strong

Evidence of the Host Organisations' support and commitment to other early career researchers.
Strong

The track record of academic department in the research area being proposed.	Strong

The suitability of the proposed training to the needs of the project.	Strong

The extent to which the proposed practice activities will support the development of the applicant as a practitioner academic.
Strong

The extent to which the training and development plan will support the practitioner academic career aspirations of the applicant.
Strong

The suitability and experience of the proposed academic support team, and their appropriateness to the development of the applicant's practitioner academic career.
Strong

The suitability and experience of the proposed practice support team, and their appropriateness to the development of the applicant's practitioner academic career.
Strong

**Score (1-5) 5: EXCELLENT 4: VERY GOOD 3: SATISFACTORY 2: UNCOMPETITIVE 1: POOR**
5

**Comments**
The support available for the candidate is appropriate and strong, based on the information provided, the candidate should be well supported.

**Significant issues that need clarifying at interview (if relevant)**

Please complete the following using the Grading system described below

**Grade**	**Criteria**	**Descriptor**	**Outcome**
A	Fully meets the criteria in all areas assessed	Excellent candidate and application	Interview / Shortlist
B	Meets all the assessment criteria with minor weaknesses	Very good candidate and application	Interview / Shortlist
C	Reasonable quality applicant however does not adequately meet the criteria	Reasonable candidate and application	Reject
D	Not competitive	Poor candidate and application	Reject

**Grade**	A

ICA ACAF R1 (2022)

Shortlisting Review Form

**Application Reference**	NIHR302864
**Application Title**	IMPACT : Interventions to improve Mental health Provision in children And young people with Chronic rheumaTological conditions
**Applicant**	Dr Polly Livermore
**Applicant Organisation**	Great Ormond Street Hospital For Children NHS Foundation Trust
**Total Amount Requested**	£604,985.00
**Reviewer Number**	

NIHR | National Institute for Health and Care Research

Summary

**Reference Number**	NIHR302864
**Lead Applicant**	Dr Polly Livermore
**Research Title**	IMPACT : Interventions to improve Mental health Provision in children And young people with Chronic rheumaTological conditions

Our purpose for collecting this data is to communicate with you about your application and have the necessary information to evaluate you for a grant. The data we collect here is collected in the public interest. Information provided here may be subject to Freedom of Information requests.

The NIHR Academy is part of the Department for Health and Social Care (DHSC), National Institute for Health and Care Research (NIHR). The contracting agent for NIHR Academy is the Leeds Teaching Hospital Trust (LTHT). The DHSC is the Data Controller and LTHT is the Data Processor under the General Data Protection Regulation (GDPR) EC 2016/679. DHSC NIHR respects the privacy of individuals who share their data and processes it in a manner that meets the requirements of GDPR. The DHSC Data Protection Officer can be contacted by email at: data_protection@dhsc.gov.uk

The NIHR privacy policy includes further information including ways we may use your data, our contact details and details on your individual rights regarding how your data is used. Your data may be shared across the NIHR, including with other coordinating centres, to allow the application to be managed and for statistical analysis, and with external grant reviewers as part of the process for managing the allocation of a grant. This notice is under constant review and will be updated and / or revised based on that review as appropriate.

Table Of Contents

1. External Peer Review

1. External Peer Review

Research Proposal

This proposed project targets a very important but frequently over-looked group of CYP, those with rheumatological conditions, and addresses the priority topic of improving their mental health. Rheumatological conditions tend to affect more girls than boys. Girls have poorer mental health than boys e.g., more girls self-harm. This proposed project is therefore important in aiming to improve CYP's poor mental health generally by targeting a specific sub-group.

This planned project focuses on parents rather than CYP. The applicant explains this decision was made following patient/public involvement which identified parental need as being the priority for action, rather than the needs of CYP as initially thought. As parents frequently seek information from the internet to help them better support their child, it makes sense that this research proposes development of a virtual assistant for use by parents especially as current parental support is inequitably distributed and accessed.

Overall, this two-phased study with an emphasis on co-design, and underpinned by the MRC Framework, seems appropriate. At times the proposal could be more congruent and specific in its narrative. For example, the project title broadly refers to improving mental health provision but the systematic review (SR) research question refers to interventions improving parental support yet does not define this or refer specifically to mental health. The Gantt chart shows WP1-3 happening in a linear fashion however, qualitative research is iterative and delays in recruitment generally mean that realistically some time overlap between these steps is likely.

Phase 1: The SR will follow the relevant methodology and report using PRISMA but more detail is needed such as defined inclusion criteria of relevant studies. Focus groups (FG) are appropriate in this context and there is flexibility for these to be online if required. More detail was needed regarding wider participation such as would FG participants be offered travel expenses and would groups be offered at different times to enable working parents to attend? 1-1 interviews would be offered for those who do not have English as a first language, would these also be offered to women/girls from cultures where participation in a mixed gender group would be considered inappropriate? The study will recruit across the UK which is good as a range of participant perspectives are needed and this would reflect the applicant's earlier point about inequity of existing support. However, would diversity of location be addressed e.g., by recruiting participants from remote/rural locations not just those in urban areas? As the age of consent is not 18 UK-wide, some CYP may give their own consent.

Phase 2: Prototype intervention development – this seems appropriate overall but digital product development is not this reviewer's area of expertise; comment can therefore only be made on general points. In several places more depth of detail was needed to better understand the research plan. For example, Work Package (WP) 3 does not specify who would be involved in the workshops to identify content (parents or professionals) and there is no mention of how participant diversity would be ensured in WP4. It is unclear why WP4 has expected levels of parental digital access. This is potentially a barrier to participation and could result in the loss of valuable data e.g., some participants might only use the VA once in a cycle but, find it really useful. WP5 methods section needed more detail to enable fuller assessment of outcomes.

This is a complex ambitious multi-staged proposal and within a limited word count it is difficult for an applicant to fully convey what they plan to do however this proposal shows a commitment to user involvement/co-design and targets the needs of a group of CYP who are often over-looked by others. The applicant will be working with an excellent team of senior researchers as well as clinical nursing colleagues and those in the charitable sector. The project aims to develop an innovative digital product which could help parents better support their child living with a long-term physical condition which also adversely affects their mental health/well-being.

Issues that could be explored at interview

- To explicitly determine the project's specific focus – is it mental health provision or parental support more generally? Could an alternative title e.g., one referring to a virtual assistant better reflect the specific focus of this proposed project?
- Ethical issues need further discussion e.g., the applicant plans to recruit via a professional group she leads – what ethical implications might this have? Would the applicant avoid recruiting FG participants from her clinical area to prevent any conflict of interest? What is the exact process for consenting parents and CYP – when and how will this take place? The budget refers to getting ethical approval for multiple sites across the UK. This could be potentially very challenging, time consuming and increase staff costs. Is there an alternative to using NHS recruitment perhaps a social media campaign could attract a broader range of recruits other than those using charity services?
- The Working Group is made up of CYP, parents and health-professionals, with UK representation of rheumatological conditions, across age and gender. What about race, ethnicity and differing parental

income/education? How would the research ensure the views of marginalised groups are included e.g., CYP who are not looked after by their parents?
- Phase 2: WP3: prototype – who are the workshop participants? Are these professionals or parents and how will they be recruited to ensure diversity? How/where will the workshops be delivered? How will participation be encouraged e.g., how would busy professionals or parents be enabled to attend?
- Phase 2: WP4 – 25 parents/carers will be recruited for up to 4-cycles of user testing. Mostly participants will be naïve to each cycle. This could be a challenge in terms of recruitment would cycles only run with 25 parents/carers or could they run with less? Could recruitment be opened up more generally across the UK e.g., through social media? Parents/carers will be requested to use the VA for 3 weeks, during which time they will be expected to access it at least 6 times, for 5 minutes duration each time. Why expect parents to use it a certain number of times? Would there not be benefit in seeing how often parents used it and for how long? Specifying exact times/number of uses does not let parents use it in the ways they need to, e.g., 2-3 minutes at a time. Could parents be involved in informing expected usage limits rather than these being set by the researchers?
- WP4 inclusion criteria – as noted, setting limits on expected parental use could be a barrier to participation, resulting in attrition and loss of data. Why can parents not just report freely on how often they used the VA, for how long, if at all? Could the content include resources for mental health support for parents or CYP or signposting to mental health charities?
- WP5 – what about reporting on what specifically the users engaged in and found helpful? More detail is needed about the qualitative element e.g., why interviews and not FG? Why only the last cycle participants? Product development is incremental and iterative, could cycle 1 participants comment on the final version of the VA compared to version 1? Are there other measures that need considered addressing health literacy? Can the applicant expand on what they plan for WP5? In particular, how can they be sure that the product is acceptable and usable by all and not just a sub-group of white, affluent families living in urban areas who are already in a better position to access support and information?
- If successful, how could the applicant contribute to the wider field of child health nursing (see below), not just paediatric rheumatology.

Any other comments

UK child health nursing research has been at a relatively low base compared to that in the much larger fields of adult and mental health nursing. The applicant states she is currently the only paediatric rheumatology nurse with a PhD however, across the UK there are very few child nurses in any speciality with doctoral qualifications. Dr. Livermore stands out amongst child health nurses in the UK not just because of her PhD but also her strong and developing research record. Her application also needs to be seen in this wider nursing context. In my opinion, she is on a pathway to becoming one of the UK's foremost child health nurse researchers and, as such, awarding her this fellowship would be a big step in supporting the development of UK child health nursing research generally. Dr. Livermore's CV shows the significant and sustained contribution she is making in paediatric rheumatology care. Primarily her proposal intends to improve patient care but she also has real potential to impact professionally on child health nursing. If she was awarded this fellowship, it would be beneficial if she considered how she could maximise her research impact beyond rheumatology to raise the profile of child health nursing research by, for example, embedding her research in pre-registration teaching, acting as a role model for clinical/academic careers for students beyond GOSH and inspiring the next generation of researchers in this nursing field. Whilst there are areas of her proposal that need clarification or refinement, overall, if she was successful her research would benefit not just patient care but UK child health nursing too. Given the paucity of child health nurses with doctorates if the candidate is successful, please can she consider developing her links with child health nurse education within her training and development plan. I enjoyed reading this submission, the principles of this proposal are strong, the candidate's CV is strong and I wish her success with her application.

Please complete the following using the Grading system described below

Grade	Criteria	Descriptor	Outcome
(A)	Fully meets the criteria in all areas assessed	Excellent candidate and application	Interview / Shortlist
(B)	Meets all the assessment criteria with minor weaknesses	Very good candidate and application	Interview / Shortlist
(C)	Reasonable quality applicant however does not adequately meet the criteria	Reasonable candidate and application	Reject
(D)	Not competitive	Poor candidate and	Reject

	application

**Grade**	B

NIHR | National Institute for Health and Care Research

Summary

**Reference Number**	NIHR302864
**Lead Applicant**	Dr Polly Livermore
**Research Title**	IMPACT : Interventions to improve Mental health Provision in children And young people with Chronic rheumaTological conditions

Our purpose for collecting this data is to communicate with you about your application and have the necessary information to evaluate you for a grant. The data we collect here is collected in the public interest. Information provided here may be subject to Freedom of Information requests.

The NIHR Academy is part of the Department for Health and Social Care (DHSC), National Institute for Health and Care Research (NIHR). The contracting agent for NIHR Academy is the Leeds Teaching Hospital Trust (LTHT). The DHSC is the Data Controller and LTHT is the Data Processor under the General Data Protection Regulation (GDPR) EC 2016/679. DHSC NIHR respects the privacy of individuals who share their data and processes it in a manner that meets the requirements of GDPR. The DHSC Data Protection Officer can be contacted by email at: data_protection@dhsc.gov.uk

The NIHR privacy policy includes further information including ways we may use your data, our contact details and details on your individual rights regarding how your data is used. Your data may be shared across the NIHR, including with other coordinating centres, to allow the application to be managed and for statistical analysis, and with external grant reviewers as part of the process for managing the allocation of a grant. This notice is under constant review and will be updated and / or revised based on that review as appropriate.

Table Of Contents

1. External Peer Review

1. External Peer Review

Research Proposal

This research proposal is ambitious, but exceptionally well thought-out and well-suited to the applicant, Dr. Livermore, and the research team Dr. Livermore has assembled. The study aims to determine parent, CYP, and health professionals preferences for an Intelligent Virtual Assistant (IVA), co-develop the IVA with multiple stakeholders, and determine acceptability and usability of the program for parents of CYP with rheumatologic conditions. This line of research addresses a critical area of study in a vulnerable population of youth and has the potential to be highly impactful to patient care and wellbeing. The use of digital health to provide support and address unmet social, emotional, and practical needs in CYP with rheumatic conditions is innovative and well thought out.

Strengths of the proposal include the use of an established EBCD methodology, an already formed steering group of CYP/parents/healthcare providers, strong mentorship group to help guide research with a broad range of expertise to support research activities, and an excellent research environment, not to mention an exceptionally strong applicant with a clear vision for how this study will help advance knowledge and patient care, as well as their own research career.

The training and development program proposal is very strong overall. Plan for an observership at SickKids in Toronto with Dr. Andrea Knight is an excellent idea and likely to be highly valuable. It is not as clear how Intro to Health Economics class will fit in as well with Dr. Livermore's career aspirations and research plans.

Recruitment of participants is planned primarily through charity and national professional networks. To the applicant's credit, some thought has been taken to consider that this may lead to a biased sample of highly-motivated and highly-engaged parents and families and recruitment will additionally be supported through clinical settings. However, there could be more intentional recruitment of high-risk vulnerable families - socially/ financially disadvantaged, those struggling with family stressors and/or mental health problems. It seems that these CYP may most benefit from the intervention and it may be worth targeting high-risk populations for work in development and design of the program. Related to this, while there is mention that flyers will be made available in other languages, there are not specific plans spelled out about which languages and/or how they will determine which languages are necessary to develop additional recruitment materials.

The biggest concern with regards to the proposal is that 6 months for the intervention development seems very short, and it is unclear how the research will progress if the team cannot produce an intervention within that time frame. However, the strengths of the environment and expertise of the team increase the likelihood of success.

Issues that could be explored at interview

-Thoughts about how to ensure that the team successful builds the new software and tests it in the limited time frame within the 3 year award.

-Alternate plans if there are problems developing the digital technology.

-Generalizability of the research; what are the potential pitfalls with regard to reaching out to the most highly vulnerable populations, will this intervention potentially worsen health inequities -- is it being designed in partnership with a diverse group of CYP/parents who are well representative of the targeted clinical population or those who are most engaged and most likely to be financially and socially well off?

Any other comments

Dr. Livermore is a highly deserving candidate who has dedicated their career to the care of CYP with rheumatologic diseases and is an established clinical leader and already well on their way to establishing themselves as an important researcher in the field. Dr. Livermore will spend 25% of their WTE on professional development.

Please complete the following using the Grading system described below

Grade	Criteria	Descriptor	Outcome
(A)	Fully meets the criteria in all areas assessed	Excellent candidate and application	Interview / Shortlist
(B)	Meets all the assessment criteria with minor weaknesses	Very good candidate and application	Interview / Shortlist
(C)	Reasonable quality applicant however does not adequately meet the criteria	Reasonable candidate and application	Reject
(D)	Not competitive	Poor candidate and application	Reject

**Grade**	A

NIHR | National Institute for Health and Care Research

Summary

**Reference Number**	NIHR302864
**Lead Applicant**	Dr Polly Livermore
**Research Title**	IMPACT : Interventions to improve Mental health Provision in children And young people with Chronic rheumaTological conditions

Our purpose for collecting this data is to communicate with you about your application and have the necessary information to evaluate you for a grant. The data we collect here is collected in the public interest. Information provided here may be subject to Freedom of Information requests.

The NIHR Academy is part of the Department for Health and Social Care (DHSC), National Institute for Health and Care Research (NIHR). The contracting agent for NIHR Academy is the Leeds Teaching Hospital Trust (LTHT). The DHSC is the Data Controller and LTHT is the Data Processor under the General Data Protection Regulation (GDPR) EC 2016/679. DHSC NIHR respects the privacy of individuals who share their data and processes it in a manner that meets the requirements of GDPR. The DHSC Data Protection Officer can be contacted by email at: data_protection@dhsc.gov.uk

The NIHR privacy policy includes further information including ways we may use your data, our contact details and details on your individual rights regarding how your data is used. Your data may be shared across the NIHR, including with other coordinating centres, to allow the application to be managed and for statistical analysis, and with external grant reviewers as part of the process for managing the allocation of a grant. This notice is under constant review and will be updated and / or revised based on that review as appropriate.

Table Of Contents

1. External Peer Review

1. External Peer Review

Research Proposal
The proposal addresses an important problem. The project aims to develop a digital service that will guide parents through ways of supporting their children at home for their mental health needs. This service will be a 'virtual assistant' (like a robot) that parents can have on their phone or on a website and will teach them about the conditions and give them ideas to help their children.<br><br>Though the project addresses an important need, the exact novelty and research questions are vague. There has been a lot of work already being carried out in this domain and by adding another application will not be ground-breaking or state of the art. Precise technical novelty as well as challenges in designing and developing the service have not been established.

Issues that could be explored at interview
There is no technical novelty. What is there in the application that does not exist already?<br><br>The research questions are vague and do not reveal any major innovation or challenge.<br><br>The state of the art is not well defined. Looks like this is simple reinvention of the state of the art.

Any other comments

Please complete the following using the Grading system described below

Grade	Criteria	Descriptor	Outcome
(A)	Fully meets the criteria in all areas assessed	Excellent candidate and application	Interview / Shortlist
(B)	Meets all the assessment criteria with minor weaknesses	Very good candidate and application	Interview / Shortlist
(C)	Reasonable quality applicant however does not adequately meet the criteria	Reasonable candidate and application	Reject
(D)	Not competitive	Poor candidate and application	Reject

Grade	C

ICA ACAF R1 (2022)

External Review Outcome Meeting - Teleconference Review Form

**Application Reference**	NIHR302864
**Application Title**	IMPACT : Interventions to improve Mental health Provision in children And young people with Chronic rheumaTological conditions
**Applicant**	Dr Polly Livermore
**Applicant Organisation**	Great Ormond Street Hospital For Children NHS Foundation Trust
**Total Amount Requested**	£604,985.00
**Reviewer Number**	

NIHR | National Institute for Health and Care Research

Summary

**Reference Number**	NIHR302864
**Lead Applicant**	Dr Polly Livermore
**Research Title**	IMPACT : Interventions to improve Mental health Provision in children And young people with Chronic rheumaTological conditions
**Total Research Cost**	604,985.00

Our purpose for collecting this data is to communicate with you about your application and have the necessary information to evaluate you for a grant. The data we collect here is collected in the public interest. Information provided here may be subject to Freedom of Information requests.

The NIHR Academy is part of the Department for Health and Social Care (DHSC), National Institute for Health and Care Research (NIHR). The contracting agent for NIHR Academy is the Leeds Teaching Hospital Trust (LTHT). The DHSC is the Data Controller and LTHT is the Data Processor under the General Data Protection Regulation (GDPR) EC 2016/679. DHSC NIHR respects the privacy of individuals who share their data and processes it in a manner that meets the requirements of GDPR. The DHSC Data Protection Officer can be contacted by email at: data_protection@dhsc.gov.uk

The NIHR privacy policy includes further information including ways we may use your data, our contact details and details on your individual rights regarding how your data is used. Your data may be shared across the NIHR, including with other coordinating centres, to allow the application to be managed and for statistical analysis, and with external grant reviewers as part of the process for managing the allocation of a grant. This notice is under constant review and will be updated and / or revised based on that review as appropriate.

Table Of Contents

1. Review

1. Review

Strengths
An innovative project clearly addressing a concern from those with lived experiences. The idea of the Virtual Assistant is one that has come from work with parents and CYP. This has clearly been informed by engagement with stakeholders including parents, carers and CYP.<br><br>The Selection Committee agreed that the candidate was strong, and supported in a good environment.

**Weaknesses**<br>Please summarise the panel's criticisms of the application, recording any suggestions for improvement.
The Selection Committee highlighted that further consideration is needed with regard to what the project focus was on, and strongly recommends that the focus on mental health is removed and to focus on issues such as quality of life, resilience and treatment agency -- Mental health appears to be the focus as presented however, in other places there was a focus on parental support. The link between overall improvement of mental health of CYPs via parental support was unclear from the information provided and thus the Selection Committee suggests that the project focuses on the parental support and how that impacts on treatment compliance and reduced conflict in the family.<br><br>It was not clear as presented how the PPI would involve a wide and diverse range of voices especially groups that are under-served.<br><br>It was also not clear from the information provided how the findings and outputs of this study would be generalisable to high risk/vulnerable groups of people, ie aspects around EDI to ensure this is adequately addressed by PPI and participants recruited.<br><br>The Selection Committee also noted that based on the application, there appears a lot to fit into the fellowship, including the technical aspects; the Selection Committee recommends that the applicant be realistic about time frames and goals at each stage.

**CONDITIONAL OFFERS ONLY**<br>Please stipulate the condition(s) to be met.<br>Please only detail conditions that were agreed by the Panel at the interview meeting.

No previous online reviews in correct status have been found.

ICA ACAF R1 (2022)

DH Decision Review Form

**Application Reference**	NIHR302864
**Application Title**	IMPACT : Interventions to improve Mental health Provision in children And young people with Chronic rheumaTological conditions
**Applicant**	Dr Polly Livermore
**Applicant Organisation**	Great Ormond Street Hospital For Children NHS Foundation Trust
**Total Amount Requested**	£604,985.00
**Reviewer Number**	
